# Longitudinal changes in mental health and the COVID-19 pandemic: evidence from the UK Household Longitudinal Study

**DOI:** 10.1017/S0033291720004432

**Published:** 2020-10-13

**Authors:** Michael Daly, Angelina R. Sutin, Eric Robinson

**Affiliations:** 1 Department of Psychology, Maynooth University, Co. Kildare, Ireland; 2 College of Medicine, Florida State University, FL Tallahassee, USA; 3 Institute of Population Health Sciences, University of Liverpool, Liverpool, UK

**Keywords:** Coronavirus infection, COVID-19, longitudinal research, mental health, nationally representative study, psychological distress

## Abstract

**Background:**

The COVID-19 pandemic has had a range of negative social and economic effects that may contribute to a rise in mental health problems. In this observational population-based study, we examined longitudinal changes in the prevalence of mental health problems from before to during the COVID-19 crisis and identified subgroups that are psychologically vulnerable during the pandemic.

**Methods:**

Participants (*N* = 14 393; observations = 48 486) were adults drawn from wave 9 (2017–2019) of the nationally representative United Kingdom Household Longitudinal Study (UKHLS) and followed-up across three waves of assessment in April, May, and June 2020. Mental health problems were assessed using the 12-item General Health Questionnaire (GHQ-12).

**Results:**

The population prevalence of mental health problems (GHQ-12 score ⩾3) increased by 13.5 percentage points from 24.3% in 2017–2019 to 37.8% in April 2020 and remained elevated in May (34.7%) and June (31.9%) 2020. All sociodemographic groups examined showed statistically significant increases in mental health problems in April 2020. The increase was largest among those aged 18–34 years (18.6 percentage points, 95% CI 14.3–22.9%), followed by females and high-income and education groups. Levels of mental health problems subsequently declined between April and June 2020 but remained significantly above pre-COVID-19 levels. Additional analyses showed that the rise in mental health problems observed throughout the COVID-19 pandemic was unlikely to be due to seasonality or year-to-year variation.

**Conclusions:**

This study suggests that a pronounced and prolonged deterioration in mental health occurred as the COVID-19 pandemic emerged in the UK between April and June 2020.

## Introduction

The emergence of the highly infectious severe acute respiratory syndrome coronavirus 2 (SARS-CoV-2) has created a global health crisis that prompted governments to execute extraordinary social distancing measures and restrictions to curtail the number of deaths caused by COVID-19. In the UK, these restrictions have had wide-ranging impacts, from limiting time outside of the home and the ability to work, to prompting the closing of childcare, and changing how and where education is delivered. An outcome of these restrictions has been a severe economic downturn causing job insecurity and unemployment (Bell & Blanchflower, 2020; ONS, 2020*a*).

There are concerns that the COVID-19 crisis has caused a tremendous amount of stress and anxiety for many (Holmes et al., 2020). The social distancing restrictions, for example, may have increased social isolation (Armitage & Nellums, 2020) and the widespread reports of the economic downturn may have caused concerns about financial insecurity (Fernandes, 2020). Given the alarmingly high recorded number of deaths caused by COVID-19, anxiety about personal health and worries about the health of family members with existing medical conditions may also be common (Shevlin et al., 2020). Because social isolation, financial insecurity, and health concerns contribute to psychological distress (Brooks et al., 2020; Lades, Laffan, Daly, & Delaney, 2020; Paul & Moser, 2009), the COVID-19 crisis is likely to be having a considerable burden on population-wide mental health.

Previous public health pandemics have been linked to increases in mental health problems. For example, the 2014–2016 Ebola outbreak is thought to have caused considerable anxiety among members of the general population in affected countries (Jalloh et al., 2018; O'Leary, Jalloh, & Neria, 2018) and there was evidence of higher prevalence of mental health problems among populations affected by the virus (Cénat et al., 2020). The 2002 SARS outbreak has commonalities with COVID-19 and there are a number of studies which suggest that aspects of psychological well-being and mental health negatively impact frontline workers and those infected with SARS (Lee et al., 2007; Su et al., 2007). However, for Ebola, SARS, and more recently the Middle East respiratory syndrome (MERS) outbreak in 2012, there was a lack of large-scale longitudinal evidence examining population-level mental health difficulties during the progression of the pandemics.

Tracking and understanding the mental health burden of the COVID-19 crisis has been identified as a public health research priority (Holmes et al., 2020). Moreover, there is a great need to understand the distribution of the mental health burden associated with COVID-19 because the social circumstances of ‘at-risk’ populations, such as older adults, the socioeconomically disadvantaged, and those with existing medical conditions, may make them particularly vulnerable to the damaging psychological effects of this pandemic (Benzeval et al., 2020; Pfefferbaum & North, 2020; Yao, Chen, & Xu, 2020). Studies to date are suggestive of declines in mental health as a result of the COVID-19 crisis (Daly, Sutin, & Robinson, 2020; Xie et al., 2020; Zhang et al., 2020). For example, a study of children in home quarantine during the outbreak of COVID-19 in Hubei province reported a higher prevalence of depressive symptoms than would normally be expected (Xie et al., 2020). Similarly, a US study reported a higher incidence of mental distress amongst a general public sample of US adults completing measures in April 2020 in comparison to a different nationally representative probability sample of US adults from the 2018 National Health Interview Survey (McGinty, Presskreischer, Han, & Barry, 2020).

Although informative, these findings may be explained by differences in sampling and measurement between the populations being compared. There is a need for longitudinal research that allows for a direct comparison of person-by-person mental health both before and throughout the duration of the pandemic using validated mental health measures. For example, a small study of young adults in Switzerland has found an increase in perceived stress and anger (but not internalizing symptoms) measured in lockdown compared to 2 years earlier (Shanahan et al., 2020) and a study of US undergraduate students found that levels of depression had increased when comparing pre-COVID-19 pandemic levels with data collected early on in the pandemic (Huckins et al., 2020). However, the extent to which these findings generalize to other groups in the population is unclear.

It is crucial that longitudinal research draw on probability-based samples drawn from across the population where the response rate is known and factors determining non-response can be accounted for (Pierce et al., 2020*a*, 2020*b*). A recent UK study examined mental health problems among UK adults participating in the UK Household Longitudinal Study (UKHLS), in which the same nationally representative sample of UK adults completed a mental health screening instrument in 2017–2019 and after the introduction of the UK government social lockdown orders on 23 March 2020 (Pierce et al., 2020*a*, 2020*b*). Compared with pre-lockdown, the prevalence of mental health problems was significantly higher in late April 2020 (approximately 1 month into lockdown) and this was particularly pronounced among females and younger age groups (Pierce et al., 2020*a*).

However, it remains unclear how these trajectories will evolve over time. For example, there is evidence that although psychological distress rose in the initial stages of the pandemic in the USA (April 2020), by June levels of distress were similar to distress levels measured pre-pandemic (Daly & Robinson, in press). Moreover, there is a need to understand how these trajectories develop for groups that may be most at risk of declines in mental health, such as those vulnerable to developing complications if infected with COVID-19 and those with pre-existing mental health conditions (Holmes et al., 2020). In the present research, we aimed to examine the extent to which mental health problems changed from before to during the COVID-19 crisis among UK adults. We made use of data from the UKHLS and examined the levels of mental health problems prior to the COVID-19 crisis and across three waves of assessment conducted between April and June 2020. Furthermore, to understand the distribution of the mental health burden of COVID-19, we tested whether changes in such difficulties have been more pronounced in key groups, including older adults, those at risk of complications due to medical conditions, those who have been previously diagnosed with clinical depression, and gender, race, education, income, and marital status subgroups.

## Methods

### Sample

We used data from the UKHLS (or *Understanding Society*) which collects high-quality longitudinal information on the economic circumstances, health, and well-being of households from across the UK. The sample comprises of a general population sample, ethnic minority boost samples, and incorporates the former British Household Panel Study (BHPS) sample into the overall sample design. All samples are probability samples where each postal address in the UK has a known non-zero probability of selection. In England, Wales, and Scotland, samples are stratified (equal probability), clustered samples of residential addresses selected from throughout the whole of the UK selected from the Postcode Address File. Northern Ireland used unclustered systematic random samples. Starting in 2009–2010 (Wave 1), eligible participants have been assessed annually through nine waves of data collection. In the UKHLS, each wave is conducted over a 2-year period and survey waves partly overlap. Detailed information on the study sampling methodology can be found elsewhere (Buck & McFall, 2011).

In this study, we utilized data from Wave 9 of the UKHLS (*N* = 32 596) that ran from January 2017 to May 2019. The household response rate (at least one member responding) in Wave 9 was 83.2% and the individual response rate was (full interview) 67.9% (Institute for Social and Economic Research, 2019). We matched this survey wave with data from three assessment waves conducted at the end of April, May, and June 2020 as part of the UKHLS COVID-19 study (Institute for Social and Economic Research, 2020). The UKHLS data are typically collected through either a self-completion online survey or through a face-to-face interview in participants' homes but moved to an online self-completion mode of data collection for the April–June COVID-19 surveys. In the 2017–2019 survey, 88.1% of participants indicated they used the Internet at least monthly, suggesting the vast majority of participants were eligible to participate.

Of those who took part in the Wave 9 survey (*N* = 32 596), 46% completed the April COVID-19 survey (*N* = 14 985) and response rates were similar amongst those issued the May (48.5%) and June (48.6%) surveys and comparable with other large-scale national surveys (Institute for Social and Economic Research, 2020; ONS, 2019). In total, 15 012 participants took part in the Wave 9/2017–2019 survey and at least one of the COVID-19 surveys and had survey weights available. Of this group, 619 were missing either 12-item General Health Questionnaire (GHQ-12) data or were excluded due to missing covariate data leaving a final sample size of 14 393 participants with 48 486 observations across the 2017–2019 and three COVID-19 survey waves. The COVID-19 survey combines the strengths of the UKHLS probability samples with inverse probability weights constructed using the rich representative Wave 9 data to allow estimates to be produced that account for unequal selection probabilities, adjust for differential non-response, and facilitate population inferences.

Survey weights were constructed using an extensive set of demographic, economic, health-related variables. Importantly, information on the mode of previous surveys was incorporated into the survey weights to help capture the likelihood participants could respond to a web survey (Benzeval et al., 2020). In addition to correcting for attrition bias by using carefully constructed survey weights incorporating known predictors of attrition, we conducted a further test for the presence of non-random attrition by examining the relationship between mental health problems in 2017–2019 and loss to follow-up in an unweighted retention probit (Fitzgerald, Gottschalk, & Moffitt, 1998). We found that mental health problems in 2017–2019 were unrelated to participation in the COVID-19 survey reducing concerns that non-random attrition may bias the outcome model.

In this study, we also examined the full UKHLS dataset (Waves 1–9 and COVID-19 study waves) including the entire set of GHQ assessments conducted from 2009 to June 2020 (*N* = 65 821; observations = 325 684) treating the survey waves as repeated cross-sections in order to estimate the population prevalence of mental health problems over the past decade and to understand recent seasonal and year-to-year changes in mental health problems.

## Measures

### Demographic characteristics

Participants reported their age, gender (male, female), and race (White, non-White including Black, Asian, and Other races), as part of the COVID-19 study and we also utilized information on the marital status and educational qualifications and household income of participants as reported in Wave 9 of the UKHLS. To examine the association between socioeconomic status and mental health problems, we examined the participant's highest level of education attainment (university degree, no degree) and net household monthly income (grouped into tertiles: ⩽£2500, £2500–£4000, ⩾£4000). Participants were grouped into one of four age groups based on their age during the pandemic: 18–34, 35–49, 50–64, and aged 65+.

### COVID-19 at-risk group

Participants were classified as in an at-risk group if they were considered clinically vulnerable to developing complications as a result of COVID-19. This was gauged by asking participants if they received communications from the NHS or Chief Medical Officer indicating they would be considered at risk of severe illness if they contracted coronavirus because of an underlying disease or health condition.

### Diagnosis of clinical depression

Drawing on data from across all study waves from 2009 to 2019, we identified whether the study participants have previously been told by a doctor or other health profession that they have clinical depression. In total, 8% of the sample reported receiving a diagnosis of this kind from their doctor.

### General Health Questionnaire-12

Mental health problems were measured using the GHQ-12 (Goldberg & Williams, 1988) which is a widely used measure of non-psychotic psychiatric cases in the general population. Participant's report the extent to which 12 symptoms are present in the past few weeks. The scale comprises items assessing anxiety/depression (e.g. ‘been feeling unhappy and depressed’, ‘lost much sleep over worry’), social dysfunction (e.g. ‘felt capable of making decisions about things?’ [reverse coded]), and loss of confidence (e.g. ‘been thinking of yourself as a worthless person’).

Participants rated the extent to which they have been experiencing each item on a four-term scale (negatively worded items scaled as 1 = ‘not at all’, 2 = ‘no more than usual’, 3 = ‘rather more than usual’, and 4 = ‘much more than usual’; positively worded items scaled as 1 = ‘better than usual’, 2 = ‘same as usual’, 3 = ‘less than usual’, and 4 = ‘much less than usual’). As in prior research (Aalto, Elovainio, Kivimäki, Uutela, & Pirkola, 2012; Goldberg et al., 1997), we use the GHQ-12 as a short screening instrument to detect probable mental health problems. We implemented the standard system of scoring to dichotomize whether participants experienced each GHQ symptom and formed a scale ranging from 0 to 12 symptoms experienced. Following accepted convention (Goldberg et al., 1997), those scoring 3 or more were termed as achieving ‘psychiatric caseness’ indicating likely risk of presenting with mental health problems. The cut-off threshold has been validated against psychiatric interviews for the detection of psychological disorders (Aalto et al., 2012; Goldberg et al., 1997).

### Data analysis

Our analyses were carried out in Stata version 15 using the *svy* commands and survey weights. We first examined within-person change in the number of symptoms reported by participants from 2017–2019 to April, May, and June 2020 using fixed-effects regression with time-invariant covariates omitted. Our main longitudinal analyses examined the presence/absence of mental health problems using weighted logistic regression models with clustered standard errors that adjusted for the statistical dependence of repeated observations on the same individuals, unequal selection probabilities, and differential non-response to each wave of the COVID-19 survey. First, we contrasted the probability of mental health problems in 2017–2019 with the April, May, and June COVID-19 survey waves in a model that adjusted for covariates. We then computed marginal effects to estimate percentage-point changes using the Stata postestimation *margins* suite of commands. This allowed the predicted marginal proportions of the binary outcome to be estimated while controlling for the distribution of covariates (Long & Freese, 2014). Changes in predicted probabilities of mental health problems were multiplied by 100 to represent percentage point changes. This analysis provided our estimate of the discrete change in the prevalence of mental health problems from 2017–2019 to April, May, and June 2020.

Next, we examined changes in mental health problems over this period for population subgroups (i.e. age groups, gender, race, marital status, education and income groups, and the vulnerability to COVID-19 dichotomous variable). To test for the presence of systematic differences in the level of change in mental health problems between population subgroups, we added interactions between the survey period dummy and each demographic/background characteristic variable. Subgroup estimates of changes over time were produced using the margins command after a logistic regression model including the relevant interaction terms. We used the Stata *lincom* command to estimate whether changes in the prevalence of mental health problems from 2017–2019 to subsequent COVID-19 survey waves differed between population subgroups. In supplementary analyses, we also gauged whether changes in mental health problems, as gauged using the GHQ ⩾3 cut-off, differed between those with/without a pre-existing diagnosis of clinical depression.

Finally, to contextualize our estimates, we examined all available GHQ data from the 12 waves of the UKHLS: waves 1–9 conducted between 2009 and 2019 and April, May, and June 2020 COVID-19 survey waves. We used weighted logistic regression analysis with standard errors clustered at the individual level to produce estimates of the percentage of the population experiencing mental health problems from 2009 to 2019 and during the COVID-19 pandemic. In addition, we used the 2009–2019 UKHLS panel data to estimate typical seasonal trends in mental health difficulties as gauged using the GHQ-12.

## Results

### Sample characteristics

The analytical sample for our longitudinal analyses included 14 393 participants (52.2% females). The sample was predominantly white (91.5%) and the average age was 50.7 (range 18–96). In total, 40.3% of the sample possessed a degree and 52.1% were married (see Table 1). In total, 7.7% of the sample were classified as at-risk/clinical vulnerability of COVID-19. The number of mental health symptoms reported increased from 1.95 (s.d. = 3.3) in 2017–2019 to 2.8 (s.d. = 3.4) in April 2020 and then declined to 2.7 (s.d. = 3.5) in May 2020 and 2.6 (s.d. = 3.6) in June 2020. Similarly, a fixed-effects regression examining within-person symptom change (see online Supplementary Table S1) showed that 0.95 (95% CI 0.85–1.05, *p* < 0.001) more symptoms were reported in April 2020 compared to 2017–2019 and the number of symptoms reported remained 0.69 (95% CI 0.57–0.81, *p* < 0.001) above baseline levels in June 2020.

**Table 1.** Sample characteristics and the prevalence of mental health problems for participants assessed in the 2017–2019 and April, May, and June 2020 waves of the UKHLS (*N* = 14 393; observations = 48 486)

Survey period	Mental health problems^a^								
	Sample characteristics	2017–2019	April 2020	Change from 2017 to 2019	May 2020	Change from 2017 to 2019	June 2020	Change from 2017 to 2019	Recovery from April to June 2020
Variable	%	%	%	%	%	%	%	%	%
Overall sample	–	24.7	37.4	+12.7***	34.6	+9.9***	31.9	+7.2***	−5.5***
Age group									
18–34 years	22.0	31.5	50.8	+19.2***	45.2	+13.7***	41.3	+9.7***	−9.5***
35–49 years	24.3	27.1	41.7	+14.7***	38.5	+11.5***	35.7	+8.6***	−6.1***
50–64 years	29.0	24.5	33.6	+9.1***	31.9	+7.4***	30.7	+6.2***	−2.9*
65+ years	24.8	15.2	27.7	+12.5***	24.2	+9.0***	21.8	+6.6***	−5.9***
Male	47.8	20.1	29.3	+9.2***	28.0	+7.9***	26.2	+6.1***	−3.1*
Female	52.2	29.1	44.6	+15.5***	40.7	+11.5***	37.0	+7.9***	−7.6***
White	91.5	24.4	37.1	+12.8***	34.2	+9.9***	31.4	+7.0***	−5.8***
Non-white	8.5	27.8	40.4	+12.6***	38.0	+10.2***	37.3	+9.6***	−3.0
Married	52.1	19.0	31.4	+12.4***	28.7	+9.7***	25.7	+6.7***	−5.7***
Not married	47.9	30.4	44.3	+13.9***	41.1	+10.7***	38.8	+8.4***	−5.5***
University degree	40.3	23.1	39.3	+16.1***	35.9	+12.8***	32.3	+9.2***	−6.9***
No degree	59.7	25.7	36.1	+10.4***	33.6	+7.9***	31.6	+5.9***	−4.4***
Income level^b^									
Bottom tertile	36.7	28.9	39.4	+10.5***	38.4	+9.5***	35.3	+6.4***	−4.1*
Middle tertile	31.2	23.6	35.5	+11.9***	32.6	+9.0***	29.6	+6.0***	−5.9***
Top tertile	32.1	20.9	36.9	+16.0***	32.2	+11.2***	30.3	+9.4***	−6.6***
COVID-19 risk	7.7	39.7	45.6	+6.1	40.0	+0.3	39.1	−0.6	−6.7
COVID-19 not elevated risk	92.3	23.4	36.7	+13.3***	34.1	+10.7***	31.3	+7.9***	−5.4***

*Note*: Estimates are derived from weighted data. Age groups are based on age reported during the COVID-19 surveys.

a Those with a GHQ ‘caseness’ score ⩾3 were classified as experiencing mental health problems.

b Net household income in the 2017–2019 wave of the UKHLS.

The prevalence of mental health problems was 24.7% at baseline and 37.4% in April 2020 during the COVID-19 pandemic; an increase of 12.7 percentage points and a 51% increase from baseline levels (see Table 1). The increase in the prevalence of mental health problems from the 2017–2019 wave to April 2020 appeared to be most pronounced amongst those in the 18–34 years old group (increase from 31.5% to 50.8%), females (from 27.1% to 41.7%), those with a degree (from 23.1% to 39.3%), and those in the top income tertile (from 20.9% to 36.9%). Those at high clinical risk of COVID-19 showed the smallest increase in mental health problems (increased from 39.7% to 45.6%). All sample characteristics and changes in the prevalence of mental health problems are shown in Table 1.

### Longitudinal change in the prevalence of mental health problems

There was a statistically significant change in the predicted probability of mental health problems from 24.3 percentage points (95% CI 23.1–25.5%) to 37.8 points (95% CI 36.4–39.2%) between 2017–2019 and April 2020 in a fully adjusted model, an increase of 13.5 percentage points (95% CI 11.8–15.1%, *p* < 0.001) or 56% from baseline levels, as shown in Table 2. Statistically significant increases in the probability of mental health problems were evident for all population subgroups between 2017–2019 and April 2020 with the exception of the COVID-19 at-risk group, as outlined in Table 2. Mental health difficulties increased by 10.3% for males (95% CI 8.0–12.5%) and by 16.4% for females (95% CI 14.1–18.7%), as shown in Table 2. Our postestimation analysis indicated this was a statistically significant difference of 6.1% (95% CI 3.0–9.3%), as shown in Table 3.

**Table 2.** Regression estimates of percentage point changes in mental health problems in the UKHLS from 2017–2019 to April, May, and June 2020 by population subgroups (*N* = 14 393; observations = 48 486)

Variable	Mental health problems^a^							
	Δ2017–2019 to April 2020		Δ2017–2019 to May 2020		Δ2017–2019 to June 2020		Recovery from April to June 2020	
	%	95% CI	%	95% CI	%	95% CI	%	95% CI
Overall	+13.5***	(11.8–15.1)	+10.4***	(8.7–12.1)	+7.6***	(5.9–9.4)	−5.8***	(−7.4 to −4.3)
Age group								
18–34 years	+18.6***	(14.3–22.9)	+13.3***	(4.4–13.2)	+8.8***	(4.4–13.2)	−9.8***	(−14.2 to −5.2)
35–49 years	+14.7***	(12.0–17.3)	+11.9***	(9.1–14.7)	+8.9***	(5.9–11.8)	−5.8***	(−8.6 to −2.9)
50–64 years	+9.3***	(6.5–12.2)	+7.7***	(4.7–10.8)	+6.2***	(3.0–9.5)	−3.1*	(−5.6 to −0.6)
65+ years	+12.4***	(9.3–15.5)	+9.2***	(6.8–11.7)	+6.7***	(3.5–9.9)	−5.7***	(−8.8 to −2.5)
Male	+10.3***	(8.0–12.5)	+8.5***	(6.1–10.8)	+6.8***	(4.4–9.3)	−3.5**	(−5.8 to −1.1)
Female	+16.4***	(14.1–18.7)	+12.2***	(9.9–14.6)	+8.4***	(6.0–10.8)	−8.0***	(−10.1 to −5.8)
White	+13.6***	(11.9–15.2)	+10.4***	(8.7–12.1)	+7.5***	(5.7–9.3)	−6.1***	(−7.7 to −4.4)
Non-white	+12.7***	(5.8–19.6)	+10.1**	(3.4–16.8)	+9.3**	(2.8–15.8)	−3.4	(−9.3 to 2.5)
Married	+13.6***	(11.6–15.6)	+10.5***	(8.6–12.4)	+7.5***	(5.5–95)	−6.1***	(−7.9 to −4.3)
Not married	+13.6***	(10.9–16.3)	+10.5***	(7.7–13.3)	+7.9***	(5.1–10.8)	−5.6***	(−8.4 to −2.9)
University degree	+16.9***	(15.0–18.8)	+13.2***	(11.2–15.2)	+9.5***	(7.6–11.4)	−7.4***	(−9.4 to −5.3)
No degree	+11.2***	(8.8–13.6)	+8.6***	(6.1–11.0)	+6.5***	(3.9–9.0)	−4.8***	(−7.1 to −2.4)
Income level^b^								
Bottom tertile	+11.2***	(8.0–14.3)	+10.3***	(6.9–13.7)	+7.0***	(3.6–10.5)	−4.2**	(−7.1 to −1.2)
Middle tertile	+12.8***	(10.0–15.7)	+9.6***	(6.8–12.3)	+6.4***	(3.5–9.3)	−6.4***	(−9.3 to −3.6)
Top tertile	+16.7***	(14.4–19.0)	+11.3***	(9.1–13.6)	+9.5***	(7.1–11.9)	−7.2***	(−9.5 to −4.8)
COVID-19 risk	+7.7	(−1.1 to –16.4)	+2.1	(−5.5 to –9.7)	−0.7	(−8.8 to 7.4)	−8.3*	(−15.4 to −12.7)
COVID-19 not elevated risk	+13.9***	(12.3–15.5)	+11.0***	(9.3–12.7)	+8.3***	(6.6–10.0)	−5.6***	(−7.2 to −4.0)

*Note:* Estimates are from marginal effects calculated after a logistic regression with standard errors adjusted for clustering at the individual-level and controlling for all characteristics presented. Age groups are based on age reported in April–June 2020 survey waves.

a Those with a GHQ ‘caseness’ score ⩾3 were classified as experiencing mental health problems.

b Net household income in the 2017–2019 wave of the UKHLS.

*p < 0.05; **p < 0.01; ***p < 0.001.

**Table 3.** Regression estimates of percentage point changes in mental health problems from 2017–2019 to April–June 2020 comparing differences between population subgroups

Variable	Subgroup differences in changes in mental health from 2017–2019 to April 2020		Subgroup differences in changes in mental health recovery from April to June 2020	
	(%)^a^	95% CI	(%)^a^	95% CI
Age group (comparison is 50–64 years)				
18–34 years	+9.3***	(4.2–14.4)	−6.7**	(−11.8 to −1.6)
35–49 years	+5.3**	(1.5–9.2)	−2.7	(−6.5 to 1.1)
65+ years	+3.1	(−1.1 to 7.3)	−2.6	(−6.7 to 1.5)
Female^b^	+6.1***	(3.0–9.3)	−4.6**	(−7.8 to −1.3)
White^c^	+0.8	(−6.2 to 7.9)	−2.5	(−8.6 to 3.6)
Married^d^	0.0	(−3.4 to 3.4)	−0.5	(−3.7 to 2.8)
University degree^e^	+5.7***	(2.7–8.8)	−2.6	(−5.7 to 0.5)
Income level^f^ (comparison is low)				
Middle tertile	+1.7	(−2.6 to 6.0)	−2.2	(−6.3 to 1.9)
Top tertile	+5.6**	(1.8–9.5)	−3.0	(−6.7 to 0.8)
COVID-19 risk	+6.5	(−2.3 to 15.3)	−2.5	(−9.7 to 4.7)

*Note:* Estimates are from marginal effects calculated after a logistic regression with standard errors adjusted for clustering at the individual-level and controlling for all characteristics presented. Age groups are based on age reported in the COVID-19 surveys.

a Those with a GHQ ‘caseness’ score ⩾3 were classified as experiencing mental health problems.

b Difference between females and males in the change in mental health problems between time points.

c Difference between whites and non-whites in the change in mental health problems between time points.

d Difference between married and non-married participants in the change in mental health problems between time points.

e Difference between those with/without a degree in the change in mental health problems between time points.

f Net household income in the 2017–2019 wave of the UKHLS.

*p < 0.05; **p < 0.01; ***p < 0.001.

Younger adults (aged 18–34) experienced an 18.6% (95% CI 14.3–22.9%) increase in risk of mental health problems whereas those aged 50–64 experienced a 9.3% (95% CI 6.5–12.2%) increase, a significant difference of 9.3% (95% CI 4.2–14.4%), as shown in Tables 2 and 3. Mental health problems increased by 5.3% more (95% CI 1.5–9.2%) in the 35–49 years old group compared to the 50–64 years old group (see Table 3). Further, socioeconomic status was associated with the rise in mental health problems. Those with a degree experienced a 5.7% (95% CI 2.7–8.8%) greater increase in mental health problems than those without a degree (see Table 3) and those in the top income tertile experienced a 5.6% (95% CI 1.8–9.5) larger increase than those in the bottom income tertile. The rise in mental health problems did not differ significantly by race, marital status, or COVID-19 risk status.

We also observed some evidence of recovery in the population prevalence of mental health problems. Our fixed-effects analyses showed that the increase in the number of symptoms reported between 2017–2019 and April 2020 was reduced by 27% (from 0.95 to 0.69) between April and June 2020 (see online Supplementary Table S1). Longitudinal analyses also revealed that mental health problems declined from a peak of 37.8% in April to 34.7% in May, and 31.9% in June 2020. Mental health problems recovered by 5.8 percentage points (95% CI 4.3–7.4%) between April and June 2020 representing a 43% decline from peak levels. All subgroups showed a decline in mental health problems between April and June 2020 (see Table 2) with the exception of non-white participants, potentially reflecting a lack of statistical power to detect changes in this group. Our regression analysis, which simultaneously adjusted for each demographic characteristic of interest, showed that the 18–34 years old group was associated with the largest decline in mental health problems (9.8%; 95% CI 5.2–14.2%), followed by being female (8%; 95% CI 5.8–10.1%) and possessing a university degree (7.4%; 95% CI 5.3–9.4%).

In supplementary analyses, we examined 8% of the sample with a pre-existing diagnosis of clinical depression. At baseline, our logistic regression analyses showed that 50.7% of this group scored above the GHQ threshold for mental health problems compared to 22.2% of other participants. However, those with a pre-existing diagnosis of depression did not experience a statistically significant increase in the prevalence of mental health problems during the pandemic (see online Supplementary Table S2) or a significant increase in symptoms (see online Supplementary Table S3). In line with the overall study results, those who had not received a diagnosis of depression experienced a marked increase in mental health problems and symptoms.

### Full UHKLS panel estimates

**Fig. 1.**
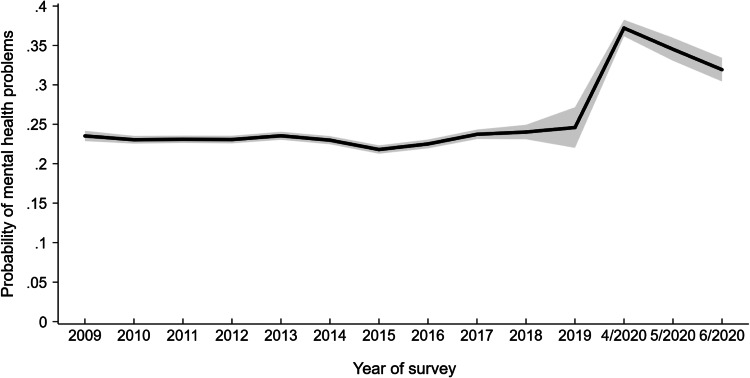
Predicted probability of mental health problems in each year of the UKHLS across nine waves of data collection from 2009 to 2019 and three waves collected in April (4/2020), May (5/2020), and June (6/2020) of 2020. Trends shown are derived from a logistic regression model with clustered standard errors (*N* = 65 821; observations = 325 684). 95% confidence intervals presented in grey. *Note*: 2019 estimate includes a reduced number of assessments (*N* = 1454).

Our initial weighted logistic regression models estimated across all survey waves and GHQ-12 assessments administered within the UKHLS (*N* = 65 821; observations = 325 684) showed that there was little change in the prevalence of mental health problems from 2009 to 2019 despite the presence of major national events such as the Great Recession and the Brexit referendum during this period (see Table 4). The percentage of mental health difficulties was highest in 2018/2019 (24.0%/24.6%) and lowest in 2015 (21.8%), as shown in Table 4 and illustrated in Fig. 1. In contrast, levels of mental health problems were markedly elevated in April 2020 (37.2%) and remained elevated in May (34.5%) and June 2020 (31.9%). Further, the role of seasonality was minimal. Our analysis of 2009–2019 data (*N* = 65 098; observations = 290 099) showed that the prevalence of mental health problems was highest in March (23.9%) and December (23.7%) and lowest in August (21.8%) in regression models that adjusted for year effects. Taken together, these analyses provide evidence that the rise in mental health problems occurring during the COVID-19 pandemic is unlikely to be attributable to typical year-to-year or seasonal variation in mental health.

**Table 4.** Regression estimates of year-to-year (2009–2020) and seasonal changes in the percentage of mental health problems in the UKHLS

Variable			Variable	Mental health problems (%)^a^	95% CI
Year	Mental health problems (%)^a^	95% CI	Month^b^		
2009	23.5	(22.8–24.2)	January	23.2	(22.5–23.9)
2010	23.0	(22.6–23.5)	February	23.5	(22.7–24.2)
2011	23.1	(22.6–23.6)	March	23.9	(23.2–24.7)
2012	23.1	(22.6–23.5)	April	23.5	(22.7–24.2)
2013	23.5	(23.0–24.0)	May	23.3	(22.5–24.0)
2014	23.0	(22.5–23.5)	June	22.7	(21.9–23.5)
2015	21.8	(21.3–22.3)	July	22.1	(21.4–22.8)
2016	22.5	(22.0–23.1)	August	21.8	(21.0–22.5)
2017	23.7	(23.1–24.3)	September	22.7	(22.0–23.5)
2018	24.0	(23.1–24.9)	October	22.9	(22.2–22.6)
2019	24.6	(22.0–27.2)	November	23.5	(22.7–24.2)
04/2020	37.2	(36.2–38.3)	December	23.7	(22.9–24.6)
05/2020	34.5	(33.1–36.0)			
06/2020	31.9	(30.4–33.4)			

*Note*. Estimates are derived from weighted data. Estimates are from marginal effects calculated after a logistic regression clustered by the individual participant identifier.

Sample for year analysis: *N* = 65 821; Obs. = 325 684 and sample for month analysis: *N* = 65 098; Obs. = 290 099.

a Those with GHQ ‘caseness’ score ⩾3 classified as experiencing mental health problems.

b Analyses examine month effects from 2009 to 2019 in logistic regression models including year fixed effects.

## Discussion

In this longitudinal population-based study, we tracked changes in mental health problems from before to throughout the COVID-19 crisis. Compared to 2017–2019, mental health problems increased markedly by over 50%, from 24.3 to 37.8 percentage points at the end of April 2020, a time when stay-at-home orders had been in place for over a month in the UK. As well as estimating the extent of the deterioration in mental health during the pandemic, we also examined the distribution of changes in population sub-groups. Although all demographics displayed increases in mental health problems, findings differed based on gender, age, and socioeconomic status. Being female and having a higher education or household income level were associated with particularly pronounced increases in mental health problems. Compared to those aged 50–64, younger adults experienced greater declines in mental health and this was particularly pronounced among 18–34 years old. Adults aged 35–49 were also at increased risk of declines in mental health (compared to 50–64 years old). In line with overall trends, both white and non-white and married/non-married participants experienced similar increases in mental health problems.

As such, our findings suggest that the mental health of a substantial proportion of the population may have been affected during the initial social lockdown phase of the COVID-19 crisis. Findings that younger adults and females showed particularly pronounced declines in mental health may reflect that these groups are known to have an underlying vulnerability to mental health problems (Weinberger et al., 2018). It is now imperative to understand the mechanisms underlying these trends. Many young adults are at the margins of the labor market and may be disproportionally impacted by the employment declines associated with the pandemic (Bell & Blanchflower, 2020; Cortes, 2020). Females may also be experiencing a disproportional burden of the economic shock associated with COVID-19. For example, in the UK, mothers in two-parent households have experienced greater increases in childcare responsibilities, interruptions to paid work, and job loss compared to fathers in such households (Andrew et al., 2020).

More participants with a university degree or high household income levels experienced an increase in mental health problems at the time of the pandemic. This finding is in line with a study of US adults which found that higher education level was associated with greater concerns about the consequences of COVID-19 (e.g. becoming seriously ill) (Sutin et al., 2020). During March–April, there were over 33 000 deaths in the UK attributed to COVID-19 and this information was widely reported in the media (ONS, 2020*b*). Higher education level may be associated with greater engagement and interest in health information (Saha, 2006), which during the current crisis may have been detrimental to the mental health of some people. It is also plausible that the COVID-19 crisis has resulted in demands that higher socioeconomic position groups are less likely to have previously experienced (e.g. experiences of job instability, childcare difficulties) compared to those of lower socioeconomic status.

Findings relating to those whose health may be most at risk because of COVID-19 were mixed. Memberships of the ‘high-risk’ medical conditions group have been advised to socially isolate in the UK and are effectively ‘shielded’ from the virus. Older age (65 years and above), but not at-risk group membership was associated with pronounced increases in mental health problems perhaps reflecting that many older adults will be aware they are at increased risk of serious illness, yet because they are not being ‘shielded’ from the virus their risk of infection remains substantial. In addition, we did not find evidence to suggest that individuals with a previous diagnosis of depression were significantly more likely to report an increase in mental health problems, instead prevalence of mental health problems (51%) remained high in this group.

Although there has been considerable media coverage of the potentially damaging effects of the COVID-19 crisis on mental health, few longitudinal studies have documented changes in mental health problems from before to during the crisis in representative samples. Studies investigating the link between the pandemic and mental health have been limited by a set of methodological shortcomings including small sample sizes (Schützwohl & Mergel, 2020), relying on the potentially biased recall of respondents to assess downturns in their mental health (Holmes et al., 2020), snowball sampling strategies implemented during the outbreak of COVID-19 (Wang et al., 2020), use of cross-sectional commercial panel surveys rather than existing probability-based longitudinal samples that better represent the general population (Twenge & Joiner, 2020), and employing short periods of follow-up to identify immediate rather than medium-term effects (Huckins et al., 2020; Pierce et al., 2020*a*).

By using data from the UKHLS probability-based samples combined with survey weights, we could ensure that the study findings were generalizable. Further, by drawing on longitudinal data, we could ensure that the mental health declines could not be attributed to differences in sampling strategies across time points. The large UKHLS sample also provided sufficient power to estimate the patterns of change in mental health problems across population subgroups including those clinically vulnerable to COVID-19. Another strength of this research is we used a well-validated mental health screening tool (GHQ-12) to assess the incidence of mental health problems in the community, rather than rely on data from those who present in healthcare settings with mental health difficulties. Finally, by utilizing three waves of assessment conducted across the duration of the UK lockdown, we could assess the persistence of the deterioration in mental health since the onset of the pandemic.

In contrast to recent findings showing relatively quick psychological adaptation to the pandemic in the USA (Daly & Robinson, in press), we found that the population increase in mental health problems showed substantial persistence in the UK. Almost 60% of the increase in the prevalence of mental health problems and over 70% of the increase in the number of symptoms reported were maintained by the end of June 2020. This persistence may reflect the severity of the restrictions imposed throughout the period of April–June 2020 and the significant health and economic threat associated with COVID-19 in the UK at this time (ONS, 2020*a*; WHO, 2020). It is also worth noting that on average our fixed-effects regression model identified an increase of just one symptom from 2017–2019 to April 2020. While this rise represents a 50% population increase in the number of symptoms reported over this period, the clinical significance of this change is unclear and likely depends on the extent to which certain individuals experienced a sharper and more sustained increase in mental health symptoms than others.

While mental health problem levels did not return to pre-COVID-19 levels, there was evidence of adjustment and coping after the initial stress of the pandemic, as the proportion of participants with mental health problems decreased from a high of 37.8% in April to 31.9% in June. The initial rise in mental health problems followed by a downward trend observed across May and June is consistent with a pattern of ‘recovery’ that is commonly observed in response to stressful or traumatic life events (Infurna & Luthar, 2018). However, as social lockdown measures continue to be eased in the UK for some, but not all (i.e. continued shielding of at-risk groups), it will be imperative to understand whether these initial changes in mental health return to baseline levels over a more prolonged period and whether there are specific population sub-groups who experience lasting psychological consequences.

As the present findings indicate that a significant number of people are likely to be experiencing mental health problems during the COVID-19 crisis, it will be important to ensure that those most at risk receive support. In particular, the increased risk of developing mental health problems among younger adults is concerning, as this is a group who may be experiencing mental health difficulties for the first time and therefore in need of early intervention. Previous research has established the substantial lifetime economic costs of mental health problems (e.g. through sickness absence and job loss) (Trautmann, Rehm, & Wittchen, 2016; MousterI et al., 2019). As such, investment in mental health treatment programs and supports is crucial, both to mitigate debilitating mental health symptoms and help maintain labor market prospects during and in the aftermath of the challenging period of the pandemic.

This research has several limitations. The response rate in the COVID-19 survey was lower than typical in the UKHLS, and although we adjusted for differential non-response through weighting our analyses, it may be the case that findings underestimate the magnitude of change in mental health problems (e.g. those experiencing declines in mental health during the COVID-19 crisis may have been more likely to have been lost to attrition). The UKHLS assesses those in private households only, meaning that those in at-risk settings such as nursing homes, prisons, and in-patient psychiatric facilities were not sampled. Our sample had few Black, Asian, and minority ethnic participants (8.5%) and it will be important for further research to identify the mental health burden associated with COVID-19 in these groups. Finally, whilst the GHQ-12 has been shown to be a valid screening instrument for assessing anxiety and depression (Aalto et al., 2012; Schmitz, Kruse, Heckrath, Alberti, & Tress, 1999), the scale does not provide a clinical diagnosis of any specific condition.

Data were collected before the COVID-19 crisis and again during April–June 2020. Because data were collected between 1 and 3 years prior to the COVID-19 crisis, declines in mental health may not be fully attributable to the emergence of the crisis. However, we drew on over 300 000 mental health assessments taken over the course of a decade (2009–2019) to show that there was little evidence of either year-to-year or seasonal changes in mental health across previous waves of the UKHLS. As such, it appeared that the size of change observed over a relatively short time span would be extremely unlikely under normal circumstances.

In summary, compared to before the emergence of the COVID-19 crisis, the proportion of adults reporting significant mental health problems increased substantially as the pandemic emerged in the UK. Further, the majority of the increase in mental health problems was sustained throughout April to June 2020. Although trends toward deterioration in mental health were observed across all demographic groups, initial declines in mental health were particularly pronounced for females, those with higher socioeconomic status, and young adults. By late June 2020, these groups showed significant improvements in their mental health but continued to experience a higher prevalence of mental health problems than prior to the pandemic.

## Data Availability

The research data are distributed by the UK Data Service and available at https://beta.ukdataservice.ac.uk/datacatalogue/studies/study?id=6614

## Supplementary material

For supplementary material accompanying this paper visit http://dx.doi.org/10.1017/S0033291720004432. click here to view supplementary material
