# Supplementary material for: Longitudinal changes in mental health and the COVID-19 pandemic: evidence from the UK Household Longitudinal Study
Source: Psychol Med. 2020 Oct 13:1–10. doi: 10.1017/S0033291720004432 (PMC7737138; doi:10.1017/S0033291720004432)
Supplement: Supplementary file 1 [file S0033291720004432sup001.docx]

**Table S1.**

Fixed effects regression estimates of within-person changes in the number of mental health symptoms reported in the UKHLS from 2017-2019 to April, May, and June 2020.

| Variable | Δ Mental health symptoms^a^ | 95% CI | p |
| --- | --- | --- | --- |
| Wave (comparison is 2017-2019) |  |  |  |
| April, 2020 | 0.95 | (0.85, 1.05) | < .001 |
| May, 2020 | 0.81 | (0.70, 0.92) | < .001 |
| June, 2020 | 0.69 | (0.57, 0.81) | < .001 |

*Note:* Estimates are from fixed effects regression models with survey weights applied and time invariant covariates omitted.

^a^ Number of GHQ symptoms experienced in the past few weeks on a scale ranging from 0-12 symptoms.

**Table S2.**

Regression estimates of percentage point changes in mental health problems from 2017-2019 to April, May, and June, 2020 for those with/without a pre-existing diagnosis of clinical depression.

|  | Mental health problems^a^ | | | | | |  | |  |
| --- | --- | --- | --- | --- | --- | --- | --- | --- | --- |
|  | 2017-2019 | | Δ 2017-2019 to April, 2020 | | Δ 2017-2019 to May, 2020 | | Δ 2017-2019 to June, 2020 | | |
| Variable | % | 95% CI | % | 95% CI | % | 95% CI | % | 95% CI | |
| Clinical depression diagnosis | 50.7 | (46.1, 55.3) | +3.0 | (-1.7, 7.7) | +2.9 | (-4.1, 9.9) | 0.0 | (-7.0, 7.1) | |
|  |  |  |  |  |  |  |  |  | |
| No clinical depression diagnosis | 22.2 | (20.9, 23.4) | +14.5*** | (12.7, 16.3) | +11.2*** | (9.5, 12.9) | 8.4*** | (6.6, 10.2) | |

*Note:* Estimates are from marginal effects calculated after a logistic regression with standard errors adjusted for clustering at the individual-level and controlling for covariates (i.e. age, sex, race/ethnicity, marital status, educational attainment, household income, high clinical risk).

^a^ Those with a GHQ ‘caseness’ score ≥ 3 were classified as experiencing mental health problems.

* *p* < .05. ** *p* < .01. *** *p* < .001.

**Table S3.**

Regression estimates of changes in the number of mental health symptoms reported from 2017-2019 to April, May, and June, 2020 for those with/without a pre-existing diagnosis of clinical depression.

|  | Mental health symptoms^a^ | | | | | |  | |  |
| --- | --- | --- | --- | --- | --- | --- | --- | --- | --- |
|  |  | | Δ 2017-2019 to April, 2020 | | Δ 2017-2019 to May, 2020 | | Δ 2017-2019 to June, 2020 | | |
| Variable |  |  | b | 95% CI | b | 95% CI | b | 95% CI | |
| Clinical depression diagnosis | |  | 0.25 | (-0.12, 0.63) | 0.32 | (-0.11, 0.74) | 0.28 | (-0.19, 0.76) | |
|  |  |  |  |  |  |  |  |  | |
| No clinical depression diagnosis | |  | 1.01*** | (0.91, 1.11) | 0.85*** | (0.74, 0.96) | 0.72*** | (0.61, 0.84) | |

*Note:* Estimates are from separate fixed effects regression analyses conducted for those with/without a diagnosis of clinical depression.

^a^ Number of GHQ symptoms experienced in the past few weeks on a scale ranging from 0-12 symptoms.

* *p* < .05. ** *p* < .01. *** *p* < .001.
